# Inflammatory Fibroid Polyp of the Esophagus: A Report of a Rare Clinical Case

**DOI:** 10.7759/cureus.77716

**Published:** 2025-01-20

**Authors:** Adam Mylonakis, Anastasios Stofas, Gregorios Paspatis, Panagiotis Sakarellos, Dimitrios Schizas

**Affiliations:** 1 First Department of Surgery, Laikon General Hospital, National and Kapodistrian University of Athens, Athens, GRC; 2 First Department of Pathology, National and Kapodistrian University of Athens, Athens, GRC; 3 Department of Gastroenterology, Venizeleio General Hospital, Heraklion, GRC

**Keywords:** endoscopy, esophageal neoplasms, esophagectomy, inflammatory fibroid polyp, polyp

## Abstract

An inflammatory fibroid polyp (IFP) is a benign, non-metastasizing tumor that can arise throughout the gastrointestinal tract, with the esophagus being an extremely rare location. We present a case of a 43-year-old male patient referred to our department following the detection of an esophageal mass during the investigation of an episode of anemia-induced loss of consciousness. Upper gastrointestinal endoscopy revealed a 3 cm distal esophageal mass protruding through the lower esophageal sphincter into the lesser curvature of the stomach. Resection of this mass was performed through an Ivor-Lewis esophagectomy, and pathological examination confirmed an IFP of the lower esophagus. The patient made an uneventful recovery and was discharged on the seventh postoperative day. This case highlights the importance of considering IFPs in the differential diagnosis of esophageal lesions and the need for individualized treatment based on patient characteristics and surgeon experience.

## Introduction

An inflammatory fibroid polyp (IFP) is an uncommon intramural reactive lesion of myofibroblastic nature that can arise throughout the gastrointestinal (GI) tract [1]. They are composed mainly of loose connective tissue and vessels with an eosinophilic inflammatory component and are not associated with dysplasia or malignancy [2].

IFP mainly affects the stomach in two-thirds of all cases (67%) followed by the small bowel (21%) and the colon (8%), while the rest of the alimentary tract is rarely affected (<3% in total) [1]. Esophageal IFP is an extremely rare lesion with 16 other cases described in the literature [3]. Although they are non-neoplastic lesions, esophageal IFPs can cause clinical symptoms due to their size and location. They typically present with dysphagia, which is usually progressive, initially with solids and subsequently with liquids as the mass increases in size [4]. The treatment of choice for esophageal IFPs is endoscopic or surgical resection, depending on the size and location of the lesion [5]. The prognosis of esophageal IFPs is generally favorable, with few cases of disease recurrence reported in the literature [3]. This case aims to present a case of an esophageal IFP and contribute to the limited literature on this rare entity, aiding clinicians in diagnosis and treatment planning.

## Case presentation

A 43-year-old man was referred to our institution following the detection of an esophageal mass during the initial investigation of anemia-induced loss of consciousness. The patient's medical history included a laparoscopic cholecystectomy for cholelithiasis and a 20-pack-year smoking history. Physical examination of the chest and abdomen was unremarkable, and routine hematological and liver function tests were within normal ranges.

**Figure 1:**
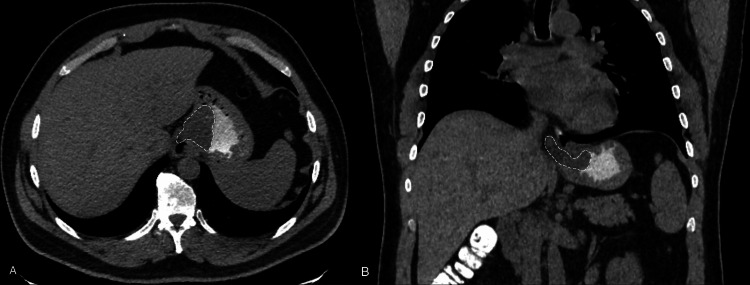
Axial (A) and coronal (B) abdominal CT scan: the highlighted area demonstrating a well-defined mass in the distal esophagus protruding into the lesser curvature of the stomach.

Upper GI endoscopy demonstrated a 3 cm ulcerating polypoid mass in the distal esophagus protruding through the lower esophageal sphincter into the lesser curvature of the stomach. A contrast-enhanced computed tomography (CT) scan revealed a well-defined mass in the distal esophagus protruding into the stomach (Figure 1). Pathological examinations of biopsies taken from the polyp raised suspicion of mesenchymal alteration, with no evidence of malignancy. Differential diagnoses included leiomyoma, GI stromal tumor (GIST), which could present with overlapping histopathological features, and fibrovascular polyp.

After a multidisciplinary team (MDT) discussion, surgical resection of the esophageal polyp was planned. The patient underwent an Ivor-Lewis esophagectomy via midline laparotomy and right thoracotomy with intrathoracic anastomosis. The postoperative course was uneventful, and the patient was discharged on the seventh postoperative day.

**Figure 2:**
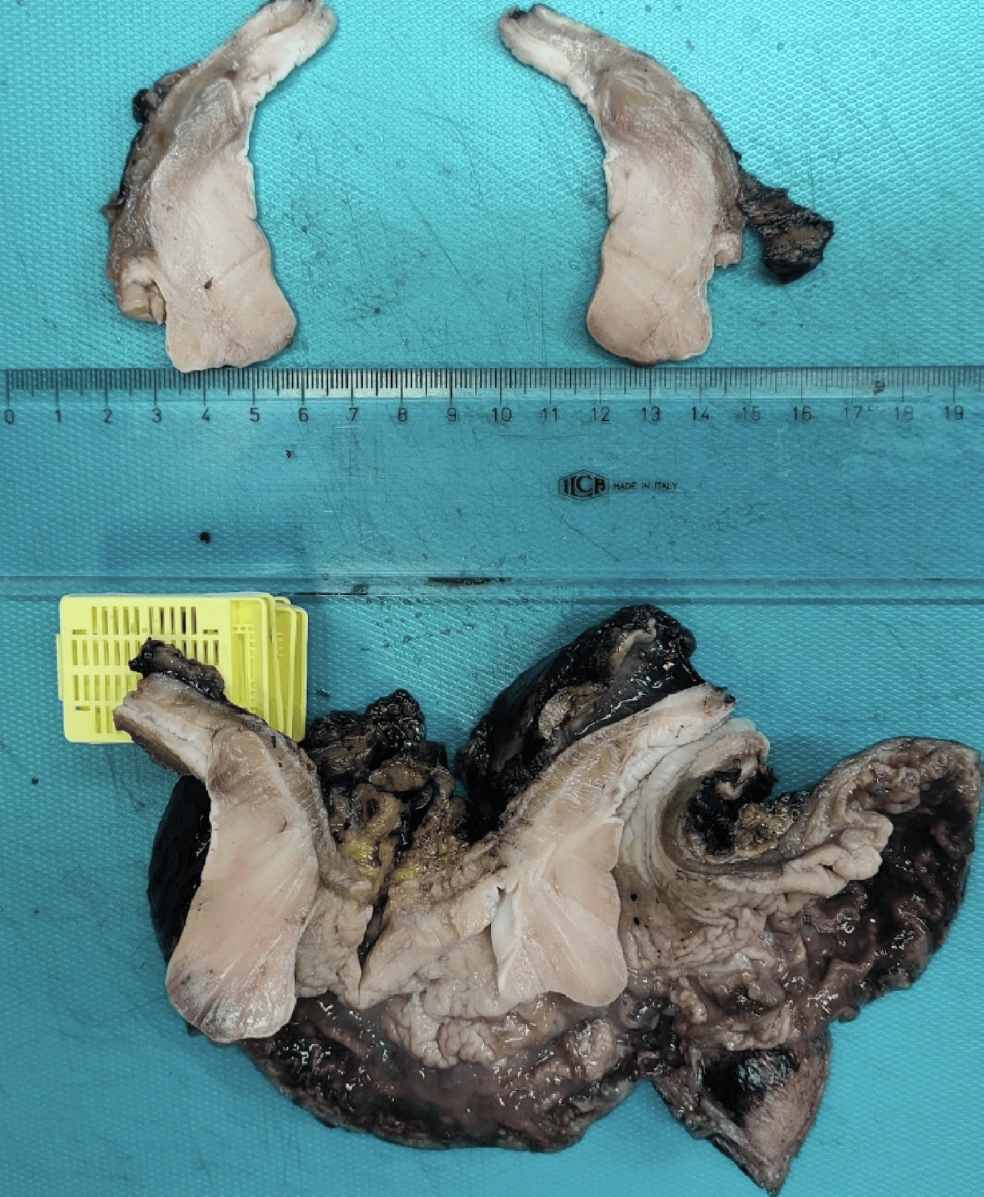
Macroscopic examination of the resected specimen, showing a fleshy, whitish tumor arising in the esophagus just above the esophagogastric junction, measuring 5.7 cm in maximum diameter, protruding into the stomach.

Histopathological examination of the resected specimen confirmed an IFP of the lower esophagus. The resected specimen measured 5.7 cm at its maximum diameter and consisted of soft-elastic, fleshy tissue (Figure 2).

**Figure 3:**
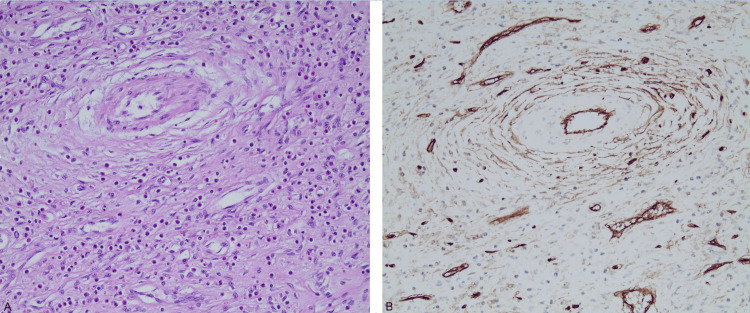
H/E staining (A) and CD34 immunostaining (B): hypocellular lesion of spindled cells with small or indistinct nucleoli and scant eosinophilic cytoplasm. The stroma is edematous with a prominent mixed inflammatory infiltrate, rich in eosinophils and lymphocytes. Small and intermediate-sized, rounded blood vessels, some with concentric (onion skin) fibrosis. CD34 immunostaining shows a whorled pattern of positive staining around some of the blood vessels.

The tumor was covered by the esophageal mucosa, which is ulcerated to a great extent. Histology showed a myofibroblastic submucosal lesion with a vascular collagenous stroma, with areas of myxoid degeneration and the presence of abundant eosinophils. By immunochemistry, the tumor showed focal whorled reactivity for smooth muscle actin (SMA) and was positive for vimentin, weakly positive for SMA, and negative for desmin, S100, KIT, and DOG-1. These findings were consistent with IFP of the gastroesophageal junction (Figure 3).

Six months after resection, follow-up CT and upper GI endoscopy showed no evidence of residual lesions or disease recurrence. The patient is scheduled for a close follow-up with endoscopy and CT scans over the next five years.

## Discussion

Esophageal IFP is a very rare benign lesion characterized by the growth of highly vascularized fibrous tissue and infiltration of various inflammatory cells [1]. To our knowledge, this is the 17th case report of esophageal IFP reported in the literature [3]. The exact pathogenesis of esophageal IFP remains unclear, but it is generally accepted to be a reactive process resulting from exposure to physical, chemical, or microbiological stimuli [6]. Clinical manifestation typically involves progressive dysphagia, along with epigastric pain and weight loss (Table 1).

**Table 1:** Clinical manifestation of esophageal IFP cases published in literature. Percentages may add up to more than 100% due to some patients experiencing multiple symptoms.

	Patients (n = 17)	Percentage
Progressive dysphagia	9	52.7%
Epigastric and retrosternal pain	5	29.4%
Weight loss	5	29.4%
Melena	3	17.6%
Vomiting	3	17.6%
Protrusion of a mass through the oral cavity	2	11.7%
Anemia	2	11.7%
Hematemesis	1	5.8%
Loss of appetite	1	5.8%
Heartburn	1	5.8%
Fever	1	5.8%

The diagnosis of esophageal IFP typically requires a multimodal approach, combining clinical evaluation, endoscopic findings, radiological imaging, and histopathological examination. Endoscopy is the cornerstone diagnostic modality, allowing direct visualization and biopsy of the lesion. Radiological techniques, such as contrast-enhanced CT, provide detailed anatomical information and help assess the lesion's size, location, and relationship to surrounding structures. Histopathology remains essential for definitive diagnosis, distinguishing IFPs from other submucosal lesions, such as leiomyomas, fibrovascular polyps, GIST, and other rare polypoid lesions of the esophagus including pseudosarcomas and carcinosarcomas [7]. Treatment options encompass both endoscopic and surgical resection, the choice of which is guided by factors such as vascularity, size, and location of the polyp [5].

The prognosis for esophageal IFPs is generally favorable postresection. However, the literature reports two instances of disease recurrence, both occurring postendoscopic resection of the polyp [5,8]. These recurrences presented one year after tumor resection and were subsequently managed with surgical excision and double antiacid administration with favorable outcomes after treatment. It is noteworthy that, in one of these cases, complete resection of the polyp was not possible due to the inability to achieve adequate submucosal lifting following a local injection at the base of the lesion. This finding suggests that inadequate resection and the persistence of reactive stimuli may contribute to disease recurrence. Therefore, an individualized treatment approach, based on both the patient's clinical features and the expertise of the surgeon and gastroenterologist, is advocated.

In our case, the patient was referred due to anemia-induced loss of consciousness. An initial upper GI endoscopy demonstrated a 3 cm ulcerating polypoid mass in the distal esophagus, which suggested a lesion of probable mesenchymal nature with no evidence of malignancy. Following an MDT discussion, the patient underwent an Ivor-Lewis esophagectomy. The postoperative histopathological examination confirmed an IFP of the lower esophagus. The patient had an uneventful recovery and remains under close follow-up with no signs of recurrence six months postresection.

## Conclusions

This clinical case highlights the importance of considering IFPs in the differential diagnosis of esophageal lesions. Due to the rarity of esophageal IFPs, a high level of suspicion and close collaboration between endoscopists, radiologists, and surgeons are necessary for proper diagnosis and management. Individualized treatment based on patient characteristics and the experience of the surgeon and gastroenterologist should be delivered to ensure the best possible outcome for the patient.
